# Stochastic dynamics and the evolution of mutations in stem cells

**DOI:** 10.1186/1741-7007-9-41

**Published:** 2011-06-07

**Authors:** David Dingli, Jorge M Pacheco

**Affiliations:** 1 Division of Hematology, Mayo Clinic College of Medicine, 200 First Street SW, Rochester, MN 55905, USA; 2 Department of Molecular Medicine, Mayo Clinic College of Medicine, 200 First Street SW, Rochester, MN 55905, USA; 3 Departamento de Matematica e Aplicações, Universidade do Minho, 4710-057 Braga, Portugal; 4 ATP-Group, Centro de Matemática e Aplicações Fundamentais, Complexo Interdisciplinar, 1649-003 Lisboa codex, Portugal

## Abstract

Stem cells are the target of mutations that can lead to life threatening diseases. However, stem cell populations tend to be small and therefore clonal expansion of mutant cells is highly sensitive to stochastic fluctuations. The evolutionary dynamics of mutations in these cells is discussed, taking into consideration the impact of such mutations on the reproductive fitness of cells. We show how stochastic effects can explain clinical observations, including extinction of acquired clonal stem cell disorders.

## 

Cancer is a consequence of multicellularity due to the fact that the cellular genome is under continuous attack from a variety of environmental or metabolic genotoxic agents. Moreover, the DNA replication machinery is not perfect [1]. Hence, the development of mutations in cells is both a natural and artificial - for example, in the case of smoking - process. The finite number of cells and the randomness of cell mutation events lead to a stochastic cell dynamics encompassing several scenarios, including extinction of acquired clonal stem cell disorders, which explains, among other things, spontaneous resolution of diseases. Although many mutations are either neutral [2] or cytotoxic, some mutations may increase the risk of malignant transformation in which the cell loses regulated growth control giving rise to a clone that may threaten the life of the organism [3,4]. The risk of acquiring mutations depends on the mutation rate, the population of cells at risk, and the average lifetime of the cell since it is unlikely that multiple simultaneous mutations occur in the same cell [5,6]. Tissues have evolved an architecture where most cells have a relatively short lifetime and undergo continuous turnover, and this mitigates the accumulation and retention of mutant cells [7]. At the root of this process are the stem cells that are able to maintain tissue integrity because of a dual phenotypic characteristic: self-renewal and production of progeny that can differentiate into various cell lineages that together constitute tissues and organs. One can visualize tissues as having a tree-like organization of cells with stem cells at one extreme and mature, non-dividing cells at the other extreme [8]. Intermediate cells divide, often at relatively high rates, but live for relatively short periods of time. Although mutations can occur at every level of this cell hierarchy, the relatively short lifetime of more mature cell stages means that, in effect, the real risk of long-lasting oncogenic mutations is restricted to the small population of stem cells and early progenitor cells that maintain a given tissue. This, in turn, effectively reduces the probability of the occurrence of mutations, given the small population of cells at risk, despite the fact that a mutation arising in a stem cell can persist for a long time. It is important to point out that the relevance of a mutation is cell context-dependent - a mutation in a gene that is not expressed in a cell is of no consequence to that cell but expression of the gene in more committed cells, downstream of the cell that is the source of the mutation, may lead to a phenotype associated with disease [9,10]. The natural history of such mutations is the focus of this article. We put forward a possible role of stochastic effects on the generation and fate of mutations acquired by stem cells. Other investigators have also explored the impact of randomness on the fate of tumor cells [11,12]. We will provide some examples from several well-known blood disorders to illustrate the concepts that will be discussed.

## Stochastic dynamics of stem cells

**Figure 1:**
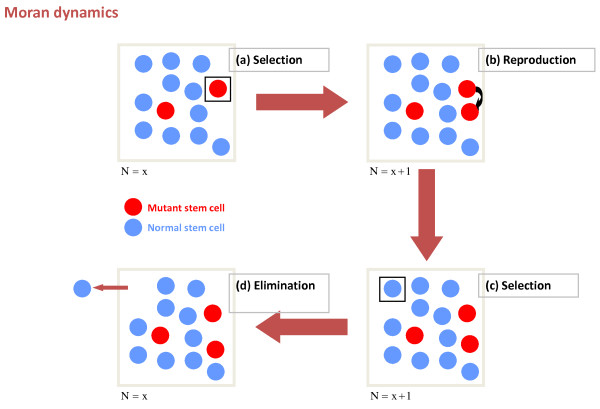
**The basic principles of the Moran process**. The Moran process assumes that, over short periods of time, the total population of cells is constant. **(a) **To start with, a cell is selected for reproduction. Selection is dependent on the frequency of the cell in the population and its reproductive fitness (*r*). **(b) **The cell divides, and the number of cells increases by one. **(c,d) **Therefore, another cell is selected for export **(c)**, which returns the population to its normal level **(d)**.

**Figure 2:**
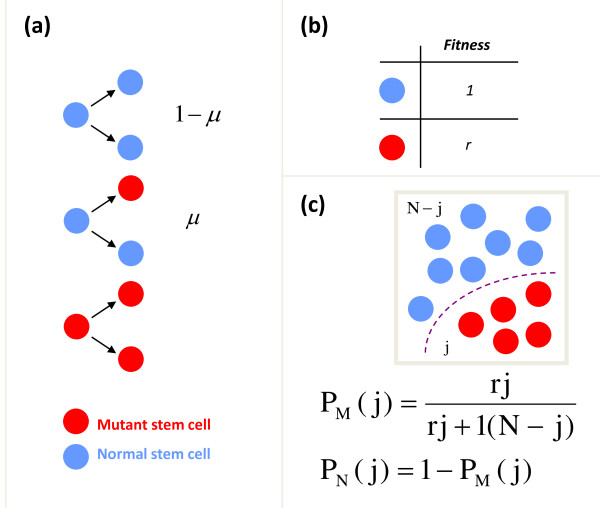
**Evolutionary dynamics under the Moran process**. **(a) **When normal cells divide, there is a probability *μ *that one of the daughter cells will have a mutation, while with probability 1 - *μ *no mutation occurs. Mutant cell replication increases the number of mutant cells - no back mutations are allowed. **(b) **Cells have a relative reproductive fitness *r *compared to normal cells, which have a fitness 1. **(c) **The probability that a cell is chosen for reproduction (*P_M _*for mutant and *P_N _*for normal cells) is dependent both on its frequency and its relative fitness. If *j *is the number of mutant cells at that time and *N *is the total number of cells present, the number of normal cells will be *N *- *j*. Since a cell has to be chosen at any time - if a mutant cell is not chosen for reproduction, a normal cell will be chosen.

**Figure 3:**
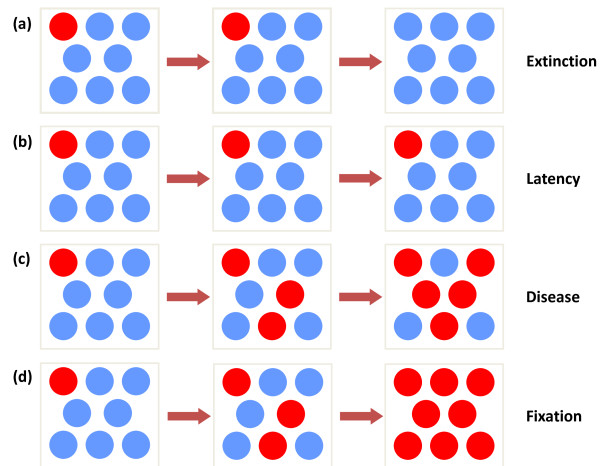
**The outcomes of Moran dynamics**. **(a,d) **Assuming that a mutant stem cell is present, stochastic dynamics will predict extinction of the mutant cell **(a) **or fixation **(d)**. **(b,c) **However, fixation may require a long time - hence the clone may persist in a latent state (no disease) **(b) **or could reach a threshold leading to a disease state **(c)**. At any of these steps, stochastic extinction is still possible, although less likely as the burden of mutant cells increases. Once the mutant clone reaches fixation **(d) **this is irreversible. Hence, the only two stable states are extinction or fixation.

For practical purposes, it is generally accepted that one can consider the number of stem cells contributing to a given tissue (for example, hematopoiesis) as constant (*N*), especially over short periods of time. As stated before, the probabilistic behavior of the finite cell population is the basis of a stochastic dynamics that can be captured by the Moran process (Figure 1). At any given time step, a cell will be selected for reproduction with a probability that is dependent on its frequency within the population and also proportional to its reproductive fitness (*r*). Reproduction will increase the net size of the population by one cell, so one must exit the pool if the population is to remain constant. It is assumed that this cell is selected randomly and that it has started the path of differentiation in the sense that such a cell will never again be selected to reproduce in the stem cell pool. Initially, we only have *N *normal cells and whenever one is selected to divide, there is a probability *μ *that one of the daughter cells will acquire a mutation in a specific gene (Figure 2a). Therefore, with probability 1 - *μ*, no mutation will occur. If a mutation occurs, there will then be a new population of (mutant) cells to consider (*M*) that can also be selected to divide. When mutant cells divide, they give rise to more mutant cells since the probability of correcting a mutation is virtually zero [5,6]. Mutations can alter the relative reproductive fitness of cells - while the relative fitness of normal cells can be defined to be one, mutant cells will have a relative fitness *r *(Figure 2a); *r *< 1 means a lower relative fitness, *r *> 1 represents a higher relative fitness and *r *= 1 means that the mutation gives no reproductive advantage compared to the remaining wild-type population. Therefore, assuming that mutant cells are present, the probability (*P_M_*) that a mutant cell is chosen for reproduction at any given time is given by:

where *j*denotes the number of mutant cells present at that time (Figure 2c). This probability will vary with time and increases as the population of mutant cells expands. Since at any time point a cell has to be chosen for reproduction, the probability of selecting a normal cell for replication is given by the equation below [13]:

If this process continues for a very long time, the result will ultimately be a state where either all the cells are normal (extinction, Figure 3a) or all the cells are mutated (fixation, Figure 3d). It is possible to calculate the probability that the mutant population will reach fixation, although the time it will take to reach this state may be very long. For example, normal hematopoietic stem cells (HSCs) on average divide once per year [14]. Given that approximately 400 HSCs are actively involved in hematopoiesis [15,16], one can consider that, on average, approximately one cell is chosen daily for reproduction [14]. Therefore, one can imagine that the process to fixation is generally slow and probably unreachable in the lifetime of a human [17] unless the reproductive fitness of the mutant is very high, a characteristic that appears to be unusual [18]. This is important - it is well known that in acquired HSC disorders such as chronic myeloid leukemia (CML), leukemic stem cells will generally coexist with normal stem cells [19]. In paroxysmal nocturnal hemoglobinuria (PNH), another clonal stem cell disorder, there is also evidence for the coexistence of normal and mutant clones [20]. Finally, many patients with acute leukemia have been cured with chemotherapy alone, a feat that would be impossible if the normal HSCs were obliterated by the malignant clone. Hence, neoplastic diseases due to acquired mutations are generally characterized by co-existence and competition of normal and mutant stem cells, and therefore the interesting dynamics occur before fixation.

**Figure 4:**
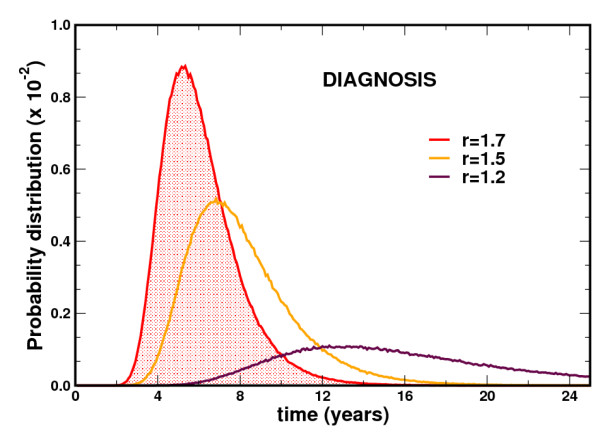
**Probability distribution functions to reach the diagnostic threshold**. Stochastic simulations of Moran dynamics, recording the probability that the mutant clone reaches the diagnostic threshold at a given time after the occurrence of the mutation (diagnosis is here defined as at least 20% of the cells being mutated) as a function of the fitness advantage (*r*) of mutated cells. The smaller the fitness advantage of mutated cells, the longer it takes for the threshold to be reached, and the smaller the probability of reaching the threshold in a given time.

In several bone marrow disorders, disease requires that a specific threshold of mutant stem cells is surpassed: at least 20% of the bone marrow cells have to be blasts to diagnose acute leukemia, and 10% clonal plasma cells are required to diagnose multiple myeloma [21]. Although these thresholds are somewhat artificial, they are meaningful at the clinical level, and they surely correlate with a corresponding burden of mutated stem-cells at the time diseases are diagnosed. We can use the Moran process to define the probability that, at least once, a mutant stem cell can expand to reach a defined threshold required to cause disease. This is again a function of the fitness (*r*) of the mutant and given by:

where *M*_0 _is the initial number of mutant cells and *M*_1 _is the number of mutant cells needed to reach the threshold [13]. This probability is independent of the size of the stem cell compartment and depends only on the relative fitness of the mutant population versus normal cells. The above model considers that the HSC pool is homogenous and cells divide symmetrically - differentiation is the result of selection of a cell to differentiate. On average, this behavior gives the same qualitative results as a more elaborate model where the symmetry or lack thereof of stem cell replication is taken into consideration. A model that captures these various dynamics has been described in detail elsewhere [22]. Assuming that a threshold has to be reached to give rise to disease, it is possible to determine the probability distribution functions for the time it will take to reach that threshold as a function of the relative fitness advantage due to a specific mutation (Figure 4). One can make two observations: (i) the probability that the threshold is reached early in time grows as the fitness advantage increases; and (ii) the width of these distributions is inversely related to the fitness advantage. For a small fitness advantage, the time distribution for reaching the threshold is very wide, rendering any summary estimates such as the average meaningless.

The Moran model assumes homogenous mixing of populations; that is, the spatial distribution of the population is not considered. This means that one cell in a specific place reproduces and a cell elsewhere is chosen for death, a scenario perhaps easiest to accept in small populations of stem cells, such as in an individual colonic crypt [23]. However, the Moran process may also be of relevance to hematopoiesis, where stem cells are distributed throughout the bone marrow, since HSCs appear to be coupled chemically and perhaps even neurologically [24,25], which may allow them to function as a homogenous population. Perhaps the best example of this tight coupling is the constant frequency of oscillations in a disease known as cyclic hematopoiesis (neutropenia). In this condition, the neutrophil count (and that of other types of cell) oscillates with a regular frequency (19 to 21 days) for the lifetime of the person [26]. For this process to be sustained, tight coupling of cellular reproduction in time must be present, even though the cells are scattered in space - otherwise the oscillations will dissipate as cell reproduction loses synchronization [10]. On the other hand, Moran dynamics introduced here does not capture the symmetric or asymmetric division of individual cells and therefore the process can only provide an average account of the population dynamics. However, one can argue that it is the population that evolves and not the individual cell(s). An analysis of the impact of the symmetry of cell division on mutant clone dynamics has been described elsewhere [22].

## Application of stochastic dynamics to disease modeling: clonal expansion

Reproduction of mutant cells leads to their expansion into a clone. How frequently the cells are chosen for replication depends on their relative frequency in the population and on their relative reproductive fitness (Figure 2b,c). Although cells with a higher relative fitness will be more likely to be selected for reproduction, it does not follow that cells with neutral or even reduced relative fitness cannot expand. Although the probability that a neutral clone will expand to reach a given threshold is small, this can happen and may lead to disease. The best example is PNH, an acquired clonal HSC disorder due to a mutation in the *PIG-A *gene. The ultimate result of this mutation is loss of expression of glycosylphosphatidylinositol (GPI)-anchored proteins from the plasma membrane of cells (for example, CD55 and CD59), which renders red blood cells unusually sensitive to complement-mediated lysis, thus leading to intravascular hemolysis and hemoglobinuria [27]. To date, no fitness advantage has been identified in cells with this mutation (summarized in [28]). We have shown that a Moran model of HSC dynamics can predict various aspects of the natural history of PNH. The model assumes that the *PIG-A *mutation gives no fitness advantage to the HSC (neutral drift). We introduced in the model (i) the number of active HSCs that are contributing to hematopoiesis, (ii) the rate of replication of the cells (approximately once per year) [16], (iii) the known mutation rate in the *PIG-A *gene [29] and an acceptable threshold of mutant cells needed to cause disease [30]. Extended simulations of the process lead to predictions of the incidence of PNH in the general population and an average clone size within patients [28] in good agreement with available data. Therefore, clonal expansion by neutral drift can lead to disease, and perhaps explain why a disease such as PNH is so rare.

Expression of *BCR-ABL *in HSCs leads to a disease resembling chronic phase CML in experimental animals, suggesting that this oncogene is enough to explain the early chronic phase of the disease [31,32]. Modeling of the dynamics in CML suggests that *BCR-ABL *does not give a fitness advantage to the mutant stem cells [33-35]. This is now supported by direct and indirect experimental evidence [36,37]. Neutral drift implies that expansion of this population of CML stem cells will generally be slow and, as a consequence, the clone size will be small. In addition, there is a reasonable chance that the mutant stem cell clone will be stochastically eliminated [34]. Can such an event happen? Currently, patients with CML are treated with tyrosine kinase inhibitors, such as imatinib or nilotinib, that have no detectable effect on the CML stem cells [38]. Therefore, it has been assumed that imatinib cannot cure patients with this disease and patients should continue therapy indefinitely. However, stochastic extinction implies that imatinib can effectively cure some patients even without directly killing the CML stem cells. In such cases, therapy could be stopped without relapse of the disease. The major issue here is how long treatment should continue since one wants to be certain that the CML stem cells have been stochastically eliminated before withdrawing therapy. This issue has been addressed using a computational model of CML dynamics under imatinib therapy that takes into consideration stochastic dynamics within the stem cell pool. Starting from diagnosis, it may take up to 5 years or more for the patient to reach a major molecular response (>4 log reduction in disease burden) and probably longer to achieve a complete molecular response. If therapy were continued for another 2 years after reaching this threshold, the probability of relapse becomes small [34] (below 2%). Recent data from the French CML group suggests that indeed some patients may be able to stop therapy without relapsing, although longer follow-up is needed [39].

## Stochastic dynamics of latency

It is not uncommon to find mutations in disease-associated genes in healthy adults. For example, almost every human has a *PIG-A *mutated clone [40]. Small clones harboring the Philadelphia chromosome and expressing *BCR-ABL *have been described [41], and the *JAK2V617F *mutation, which is normally associated with chronic myeloid neoplasms, may be present in up to 1% of the population [42,43]. The origins, implications and dynamics of these observations have been discussed elsewhere [44]. *PIG-A *mutant clones may disappear during follow up [40] or fluctuate stochastically but others may persist without detectable evolution, as has been reported in individuals with *JAK2V617F *essential thrombocythemia [45]. Latency is therefore compatible with stochastic behavior within the stem cell pool (Figure 4). However, there is at least one other scenario that can explain latency. In many if not all tumors, acquisition of the full cancer phenotype may require the serial accumulation of gain-of-function mutations in oncogenes and loss-of-function mutations in tumor suppressor genes, as well as epigenetic changes. For example, the adenoma to carcinoma sequence in colon cancer is associated with mutations in *APC*, *K-RAS*, and *TP53 *amongst others [46]. Hence, it is possible that a transient clone may arise harboring one of these mutations but, without the accumulation of additional mutations, it may not grow enough to be detectable, thus remaining a latent clone.

## Stochastic extinction

The stochastic dynamics associated with the Moran process predicts the possibility of extinction of any mutant clone, irrespective of its relative fitness. This phenomenon, however, becomes quite likely when the mutation either gives a relative fitness disadvantage or is neutral. It is important to remember that even for mutations that increase the reproductive fitness of the cell, extinction is still possible. One can computationally show that even for a mutation that increases the relative fitness of cells by a factor of two, which, in evolutionary terms, constitutes a very high fitness advantage, the probability of extinction of the mutant cell is still about 50% [13]. Of course, as the cell expands into a clone, extinction becomes less likely, explaining why most tumors do not resolve without therapy.

However, stochastic extinction is not just an *in silico *curiosity: there is evidence that clonal extinction occurs *in vivo*. Let us discuss several examples. First, healthy individuals have been shown to have small clones with the *Bcr-Abl *oncogene but not necessarily develop CML [41]. In some of these individuals, the clone will disappear in time without any specific therapy. Another example is patients who have been diagnosed with myelodysplastic syndromes, a group of clonal preleukemic stem cell disorders, that in some cases resolve without any specific therapy directed at the malignant clone [47]. One of the best examples of clonal extinction is that observed in transient myeloproliferative disorder (TMD), which occurs in a significant fraction of children with Down's syndrome and is often diagnosed at or soon after birth [48,49]. The malignant cells appear to have a very 'primitive origin', implying mutations in early progenitor cells if not in the HSCs. In the vast majority of patients, the condition resolves with minimal or no therapy, although it is lethal in some cases. One of the fascinating features about PNH is that the disease sometimes resolves in the absence of therapy (other than allogeneic bone marrow transplantation) that directly targets the mutant clone. The frequency of this spontaneous resolution is in the range of 12 to 15% and has been confirmed using both the Ham's test (a test of the sensitivity of red cells to complement-mediated lysis) and the more sensitive flow cytometry assay (which directly determines the fraction of cells that belong to the PNH clone by detection of CD55 and CD59 positive and negative cells) [50,51]. While no clear explanation has been provided for this phenomenon, our own simulations of HSC dynamics predict that clonal extinction after diagnosis can occur in about 12% of patients, in excellent agreement with epidemiological data [28].

We wish to remind the reader that, in principle, other potential explanations, alternative to stochastic extinction, could exist for the disappearance of mutant clones. For example, our model does not consider the potential impact of immune surveillance and elimination of tumor cells by the immune response. There is some evidence for this phenomenon, especially in the context of allogeneic stem cell transplantation. However, elimination of a mutant clone due to an autologous immune attack is also possible. These scenarios are not mutually exclusive and perhaps either occurs in a subset of patients.

## Conclusions

Stochastic behavior is an intrinsic aspect of life - both at the cellular and organismal level. Acquisition of mutations and the evolution of clones are processes that are highly sensitive to stochastic effects since the population under consideration is often small. Mathematically, it is as if cells play dice - many times the impact of such a gamble is inconsequential, at other times it leads to the loss of a particular cell lineage. Unfortunately, there are times when acquired mutations lead to clonal expansion and disease. Depending on the fitness of the mutant clone, stochastic extinction is possible, especially when the population is small and the relative fitness advantage minimal compared to normal cells but also even when the population is not small or the fitness advantage is significant. It is in these circumstances that chance may play a role in cure versus disease. Reduction in disease burden to low levels could, in principle, lead to clonal extinction, especially if the fitness advantage of the mutant cells can be reduced by therapy.
